# NEMA characterization of the SAFIR prototype PET insert

**DOI:** 10.1186/s40658-022-00454-2

**Published:** 2022-06-13

**Authors:** Parisa Khateri, Werner Lustermann, Christian Ritzer, Charalampos Tsoumpas, Günther Dissertori

**Affiliations:** 1 Institute for Particle Physics and Astrophysics, ETH Zürich, Zürich, Switzerland; 2 Department of Nuclear Medicine and Molecular Imaging, University of Groningen, Groningen, The Netherlands

**Keywords:** PET, PET/MRI, Dynamic imaging, Preclinical imaging, NEMA performance

## Abstract

****Background**:**

The SAFIR prototype insert is a preclinical Positron Emission Tomography (PET) scanner built to acquire dynamic images simultaneously with a 7 T Bruker Magnetic Resonance Imaging (MRI) scanner. The insert is designed to perform with an excellent coincidence resolving time of 194 ps Full Width Half Maximum (FWHM) and an energy resolution of 13.8% FWHM. These properties enable it to acquire precise quantitative images at activities as high as 500 MBq suitable for studying fast biological processes within short time frames (< 5 s). In this study, the performance of the SAFIR prototype insert is evaluated according to the NEMA NU 4-2008 standard while the insert is inside the MRI without acquiring MRI data.

****Results**:**

Applying an energy window of 391–601 keV and a coincidence time window of 500 ps the following results are achieved. The average spatial resolution at 5 mm radial offset is 2.6 mm FWHM when using the Filtered Backprojection 3D Reprojection (FBP3DRP) reconstruction method, improving to 1.2 mm when using the Maximum Likelihood Expectation Maximization (MLEM) method. The peak sensitivity at the center of the scanner is 1.06%. The Noise Equivalent count Rate (NECR) is 799 kcps at the highest measured activity of 537 MBq for the mouse phantom and 121 kcps at the highest measured activity of 624 MBq for the rat phantom. The NECR peak is not yet reached for any of the measurements. The scatter fractions are 10.9% and 17.8% for the mouse and rat phantoms, respectively. The uniform region of the image quality phantom has a 3.0% STD, with a 4.6% deviation from the expected number of counts per voxel. The spill-over ratios for the water and air chambers are 0.18 and 0.17, respectively.

****Conclusions**:**

The results satisfy all the requirements initially considered for the insert, proving that the SAFIR prototype insert can obtain dynamic images of small rodents at high activities ($\sim$ 500 MBq) with a high sensitivity and an excellent count-rate performance.

## Background

SAFIR (Small Animal Fst Insert for mRi) is a preclinical Positron Emission Tomography (PET) insert designed for a 7 T Bruker BioSpin 70/30 Magnetic Resonance Imaging (MRI) scanner [1, 2]. It has been designed following the need to measure fast biological processes such as cerebral blood flow of small rodents [3]. The ambitious goal is to achieve precise quantitative PET images of $[^{15}\hbox {O}]\hbox {H}_{2}\hbox {O}$ with a spatial resolution of ${\sim }$ 2 mm, a temporal resolution of < 5 s and a quantitative voxel accuracy better than 10%. This implies measurements with activities as high as ${\sim }$ 500 MBq for sufficient counting statistics. High rate capability in turn requires short coincidence resolving time (< 500 ps) and small coincidence time window (${\sim }$ 500 ps) in order to reduce the number of random coincidences [2, 4]. In addition to that, the detector should be designed so that the readout channels are able to handle such a high rate.

Since these requirements cannot be fulfilled by any other preclinical PET scanners [5–16], we have designed the Small Animal Fast Insert for mRi (SAFIR) system. The prototype version of SAFIR has been built and initially characterized, showing an excellent time resolution of 194 ps and an energy resolution of 13.8% [17–19]. In this study, we evaluate the SAFIR prototype performance according to the National Electrical Manufacturers Association NU 4-2008 standard [20] which we refer to as NEMA in the rest of this paper. In addition, the energy resolution and the Coincidence Resolving Time (CRT) of the scanner are studied as a function of activity.

## Materials and methods

### The SAFIR prototype PET insert

The SAFIR prototype PET insert comprises 2880 lutetium-yttrium oxyorthosilicate (LYSO) crystals arranged into 16 rings with an inner ring diameter of 128.1 mm. It covers an axial field of view of 35.6 mm. The insert is designed as a dodecagon with 12 identical sectors. Each sector hosts two detector modules. Each detector module comprises two LYSO crystal matrices, one made of $8\times 7$ and one made of $8\times 8$ crystals with ${2.12 \times 2.12 \times 13.0}\, {\hbox {mm}^{3}}$ size arranged on a grid with 2.2 mm pitch, using enhanced specular reflector foil (3M Vikuiti Enhanced Reflector Films) as spacer. Hence, the detector head in each sector is made of $2 \times 2$ parallel crystal matrices with 0.6 mm gap, aligning 16 crystals in axial direction times 15 crystals in transaxial direction. The crystal matrices are one-to-one coupled to Silicon Photo-Multiplier (SiPM) arrays (Hamamatsu S13361-2050 AE-08 SPL MPPC) with 2.2 mm pitch. Two crystal matrix and SiPM array assemblies are in turn mounted onto one detector module board hosting at the same time four PETA6SE Application-Specific Integrated Circuits (ASICs) [21].

### Data acquisition and data processing

The PETA6SE ASICs provide digitized energy and timing information of hits in the crystals, which are continuously read out by means of Field Programmable Gate Arrays (FPGAs) and transferred to the Data Acquisition (DAQ) computer via 12 optical Ethernet links (1 Gbit/s each).

The data are acquired with identical settings for the overvoltage of the SiPM arrays (6 V), the readout frequency (280 MHz) and the energy threshold (30 LSB, corresponding to 100 keV) of the PETA6SE ASIC. For all measurements, we applied a relative timing threshold of 75 LSB (corresponding to 45 mV), except for the scatter fraction and count rate measurements, where timing thresholds of 150 LSB (corresponding to 90 mV) and 250 LSB (corresponding to 150 mV) were used. This is required for the proper functioning of the SAFIR prototype at high activities up to 500 MBq.

The acquired raw data are processed off-line. We apply energy and timing calibrations converting time counter information into time stamps in picosecond and Charge to Digital Converter (QDC) values into energies in electron volt [19]. These calibrated hit data are filtered by an energy window of 391–601 keV and sorted into coincidence events using the single window method and a coincidence time window of 500 ps. This coincidence window is large enough for the largest measured diameter of 50 mm. Because, considering the time resolution of 194 ps Full Width Half Maximum (FWHM), the time difference between the furthest possible $\gamma$-rays (${\sim 170}\, {\hbox {ps}}$) falls within the coincidence window by ${4\sigma }$ (${170}\, {\hbox {ps}} + 4\sigma = {499.5}\, {\hbox {ps}}$). Coincidence events with more than two singles as well as those with a tangential angle between the singles smaller than $90^\circ$ are eliminated. This angle confines the diameter of the field of view (FOV) to ${\sim 90}\,{\hbox {mm}}$. The resulting coincidence data set is stored in list mode. The same processing parameters are applied to all data reported below. For the peak sensitivity measurement, we create a second coincidence data set, by filtering and sorting the data using a larger energy window of 250–650 keV in addition to the one mentioned above.

We use Software for Tomographic Image Reconstruction (STIR) for the image reconstruction [22]. The coincidence data sets, stored in list mode, are sorted into three-dimensional (3D) projection data, which are then reconstructed into images. We employ two reconstruction methods: (1) Filtered Backprojection 3D Reprojection (FBP3DRP) [23] and (2) Maximum Likelihood Expectation Maximization (MLEM) [24]. The voxel size is ${0.55 \times 0.55 \times 1.1}\, {\hbox {mm}^{3}}$. When we perform MLEM reconstructions, we use 30 iterations and apply a Gaussian filter with a FWHM of ${1.1 \times 1.1 \times 2.2}\,{\hbox {mm}^{3}}$ after each iteration, except for the reconstruction of the spatial resolution data, where NEMA requires a reconstruction without any filtering.

In the FBP3DRP method, a cylindrical scanner model with an equidistant spacing of crystals in axial and transaxial direction is used while in the MLEM method, the scanner is modeled with the exact generic geometry [25]. It has been shown that using a more accurate model of the scanner improves the image quality [26].

### Characterization of the SAFIR prototype insert

The performance of the SAFIR prototype insert is characterized according to the NEMA protocol while it is inside the MRI scanner. In addition, the energy resolution and the CRT of the insert are evaluated at different activities up to 537 MBq using the NEMA mouse scatter phantom. Since the assessment of MRI-compatibility is not the purpose of this work, all measurements are performed without acquiring MRI data. However, we have previously investigated the MRI-compatibility and have observed no interference during MRI acquisition [27].

#### Spatial resolution

We measure the spatial resolution using a ^22^Na point source (Eckert & Ziegler Isotope Products, MMS09-022), with an activity of 0.487 MBq and a source diameter of 0.25 mm centered in an acrylic cube of 10 mm edge length. We acquire data from the point source for two axial positions of 0.0 mm and 8.9 mm (equivalent to a quarter of the axial FOV) and for 10 radial positions from 0.0 to 45.0 mm in steps of 5.0 mm. At least $10^5$ coincidence events are collected per source position.

Although not required by NEMA, we reconstruct the images of the point source using MLEM as well as FBP3DRP without any smoothing or post-reconstruction filtering. It has been shown that prescribing Filtered Backprojection methods to measure the spatial resolution is not appropriate for scanners with a block detector geometry, because these methods do not include the scanner model, thus cause streak artifacts and degrade the spatial resolution, especially in directions perpendicular to the scanner blocks [28]. The FWHM and Full Width Tenth Maximum (FWTM) of the images are obtained according to NEMA.

#### Sensitivity

We measure the sensitivity using the same point source as for the spatial resolution measurements (“Spatial resolution” section). The source is located on the central axis of the scanner and is axially moved in steps of 1 mm starting from the axial offset of $-15$ mm and ending with the axial offset of 15 mm. We collect $8\times 10^4$ coincidence events per source position. The sensitivity at each axial position is calculated according to NEMA. The system sensitivities for mouse and rat are not calculated as SAFIR has a shorter axial FOV than required by NEMA.

#### Count rate performance and scatter fraction

We use NEMA mouse and rat scatter phantoms to measure count rate performance and scatter fraction. We use an ^18^F labeled radiotracer as the radioactive source. There is no shielding to stop $\gamma$-rays from out of the FOV. The start and end activities are 537 MBq and 0.22 MBq for the mouse phantom, and 624 MBq and 1 MBq for the rat phantom, respectively. The activities are measured using a dose calibrator (Medisystem MEDI 405), then the activity concentrations are calculated by dividing the activity by the source volume. At least $5\times 10^5$ coincidence events are collected per acquisition. Data analysis is performed according to NEMA to obtain the total, true, scattered and random count rates as well as the Noise Equivalent Count Rate (NECR) and the system Scatter Fraction (SF).

#### Energy resolution and coincidence resolving time

Using the data set collected with the mouse scatter phantom for the count rate measurement (“Count rate performance and scatter fraction” section), we evaluate as well energy resolution and CRT as a function of activity. We report FWHM of a Gaussian fit (480–580 keV) to the coincidence energy spectrum as the energy resolution. The maximum of the coincidence timing spectrum is obtained by a parabolic fit through the highest bin and its two neighbours. We measure the FWHM of the spectrum by linearly interpolating between the bins at half the maximum and report the width as coincidence timing resolution [19].

#### Image quality study

We use the NEMA image quality phantom comprising: (1) two cold chambers, one filled with water and one filled with air, (2) five hot rods of (1, 2, 3, 4 and 5) mm diameters and (3) a uniform region. We fill the phantom with an ^18^F labeled radiotracer. According to NEMA, the measurement should be done with an initial activity of 3.7 MBq and an acquisition time of 20 min. Since the SAFIR prototype insert does not cover the whole length of the phantom, we run the measurement in two bed positions and modify activity and acquisition time such that the same number of annihilations are produced in both bed positions, taking into account the decay of the activity. In the first bed position, the cold rods together with the uniform region are measured with an initial activity of 4.2 MBq for 25 min and in the second bed position, the hot rods are measured with an initial activity of 3.3 MBq for 32 min.

We reconstruct the data using MLEM with random, attenuation, scatter and normalization corrections embedded into the reconstruction algorithm. In addition, we calibrate the reconstructed image providing absolute voxel values and thus quantitative PET data. Both, data corrections and the quantitative calibration are described below.

The reconstructed images are analyzed according to NEMA to obtain the following measures:

- *Uniformity*: The mean, maximum, minimum and Standard Deviation (STD) of the counts in a cylindrical Region of Interest (ROI) of 22.5 mm diameter and 10 mm length are measured in the center of the uniform region.
- *Recovery coefficient*: The recovery coefficient and its STD are calculated for each hot rod.
- *Spill-over ratio*: The spill-over ratio and its STD are evaluated for each cold chamber.

In addition to the above-mentioned values and in order to evaluate the accuracy of the measured image, we calculate the deviation of the absolute voxel value as follows:

1 $$\begin{aligned} Deviation (\%) = \frac{E - m}{E}\times 100\% \end{aligned}$$

where $m$ is the measured mean value of the uniform region and $E$ is the expected voxel value.

We apply the following corrections to obtain quantitative PET data:

- *Random correction*: We estimate the number of random coincidences per Line of Response (LOR) using the singles-prompt method introduced by J.F. Oliver et al. [29]. This method is an extension of the singles-rate method [30], outperforming the singles-rate method at high activities.
- *Attenuation correction*: The attenuation maps of the phantoms and the bed are generated based on their known geometries, the material compositions and their corresponding attenuation coefficients taken from the National Institute of Standards and Technology (NIST) reference database [31]. The attenuation correction factors are calculated using these maps.
- *Scatter correction*: We use the Single Scatter Simulation (SSS) method implemented in STIR to estimate the number of scattered events that occurred in the phantom [32, 33]. In this method, the attenuation map and the scanner geometry are down-sampled in order to accelerate the computation. We use a down-sampling factor of two.
- *Detector normalization*: We employ the direct normalization method to obtain the normalization factors [34, 35]. A cylinder phantom of 74 mm diameter and 50 mm length uniformly filled with an ^18^F labeled tracer is located in the center of the scanner. The data are acquired with a starting activity of 141 MBq for ${\sim 14} {\hbox {h}}$, resulting in a total number of $2.65\times 10^9$ coincidences and an average number of 1768 counts per LOR. The normalization factors are obtained by correcting for different lengths of intersection between the LOR and the cylinder volume and the effects of attenuation, scatter and randoms in the collected data.
- *Quantitative calibration of voxel values*: A cylinder of 40 mm diameter and 20 mm length uniformly filled with an ^18^F labeled tracer located in the center of the scanner is measured for 30 min with a starting activity of 4.4 MBq. We reconstruct the image including normalization, attenuation, random, and scatter corrections. The average voxel value is computed inside a cylindrical ROI of 35 mm diameter and 15 mm length in the center of the image. The absolute calibration factor is calculated as the ratio of the expected number of counts per voxel to the average voxel value in the ROI.

## Results and discussion

### Spatial resolution

**Fig. 1:**
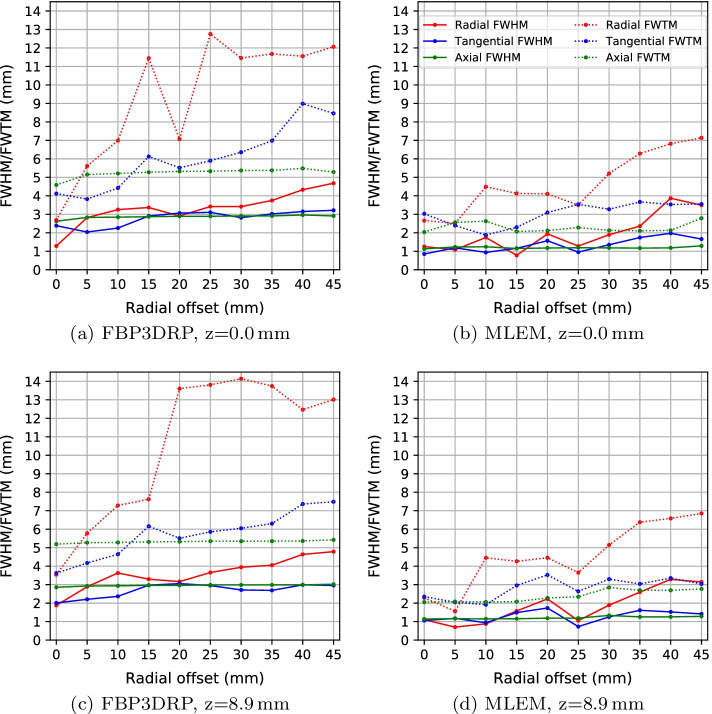
Spatial resolution in radial, tangential and axial directions for the point source located in the center of the axial FOV (**a**, **b**) and at one forth of the axial FOV (**c**, **d**). The image reconstruction methods are FBP3DRP (**a**, **c**) and MLEM (**b**, **d**)

Table 1 and Fig. 1 show the spatial resolution results for the axial positions of 0.0 mm and 8.9 mm using FBP3DRP and MLEM.

**Table 1:** Spatial resolution results of the SAFIR prototype PET insert. All values are in mm

	FBP3DRP						MLEM					
	Radial		Tangential		Axial		Radial		Tangential		Axial	
RO$^{*}$	HM$^{*}$	TM$^{*}$	HM	TM	HM	TM	HM	TM	HM	TM	HM	TM
*At the center of axial FOV*												
0	1.28	2.69	2.38	4.12	2.63	4.59	1.26	2.66	0.85	3.03	1.13	2.04
5	2.82	5.61	2.04	3.82	2.83	5.15	1.08	2.51	1.19	2.39	1.23	2.56
10	3.25	6.99	2.26	4.42	2.85	5.21	1.74	4.49	0.94	1.88	1.24	2.63
15	3.37	11.45	2.91	6.13	2.87	5.28	0.78	4.13	1.16	2.30	1.15	2.07
20	2.91	7.07	3.07	5.52	2.89	5.32	1.94	4.10	1.57	3.10	1.18	2.12
25	3.42	12.75	3.11	5.9	2.89	5.33	1.28	3.50	0.95	3.54	1.19	2.28
30	3.42	11.46	2.82	6.36	2.91	5.37	1.89	5.21	1.35	3.28	1.18	2.13
35	3.75	11.68	3.02	6.98	2.91	5.38	2.36	6.28	1.74	3.67	1.17	2.11
40	4.33	11.55	3.15	8.99	2.96	5.49	3.87	6.82	1.97	3.53	1.18	2.13
45	4.68	12.06	3.22	8.46	2.91	5.29	3.49	7.14	1.66	3.55	1.29	2.79
*At 1/4 of the axial FOV*												
0	1.88	3.54	2.01	3.62	2.86	5.19	1.10	2.29	1.06	2.35	1.14	2.05
5	2.88	5.78	2.21	4.17	2.93	5.27	0.70	1.56	1.17	2.05	1.16	2.09
10	3.63	7.28	2.37	4.64	2.94	5.28	0.88	4.45	0.94	1.92	1.15	2.06
15	3.3	7.62	2.97	6.16	2.96	5.31	1.57	4.26	1.49	2.96	1.16	2.08
20	3.16	13.6	3.06	5.51	2.96	5.33	2.23	4.45	1.73	3.53	1.18	2.28
25	3.65	13.81	2.95	5.86	2.98	5.35	1.05	3.65	0.73	2.64	1.19	2.34
30	3.94	14.14	2.71	6.05	2.98	5.35	1.89	5.15	1.25	3.30	1.33	2.85
35	4.05	13.74	2.69	6.3	2.99	5.35	2.59	6.38	1.61	3.04	1.26	2.70
40	4.64	12.47	2.99	7.36	2.99	5.36	3.29	6.58	1.52	3.35	1.26	2.69
45	4.78	13.02	2.96	7.48	3.02	5.42	3.14	6.85	1.41	3.04	1.28	2.77

*RO = Radial Offset; HM = FWHM; TM = FWTM

The MLEM algorithm yields better spatial resolution results than the FBP3DRP method. For instance, the average FWHM over radial, tangential and axial directions at the 5 mm radial offset and 0.0 mm axial offset is 2.6 mm for the FBP3DRP method and 1.2 mm for the MLEM method. Using FBP3DRP, the spatial resolution, especially degrades with respect to FWTM due to the streak artifact that is present in the reconstructed image. The FWHM in radial direction degrades toward the edge of the scanner due to the parallax effect.

We compare the SAFIR prototype results with a group of preclinical PET scanners, referred to as *reference scanners* in this paper [5–15]. Among these scanners, Hyperion II^D^ and nanoScan are for combined PET-MRI systems and Bruker is designed for the same MRI system for which SAFIR is designed. Table 2 presents the spatial resolution of SAFIR and the *reference scanners* at 5 mm radial offset. PET scanners of similar crystal size yield a slightly better spatial resolution than the SAFIR prototype when using FBP3DRP reconstruction. This is related to the geometry of the scanner. For scanners of relatively large detector heads such as SAFIR with $(8+7)\times 8$ crystals per module, the FBP3DRP method introduces error, as this method requires regularly spaced projection data which in turn requires regularly spaced detector elements. Interpolating the crystal positions into a regular space causes artifacts in the image and degrades the spatial resolution. The iterative algorithm does not require interpolation. It uses the exact *generic* geometry and thus yields better results. In addition, all scanners in Table 2 with a similar crystal size as SAFIR have a shorter crystal length, except for LabPET 8 which uses depth of interaction information and has a continuous arrangement of the crystals on a ring.

**Table 2:** Comparison of the spatial resolution of different preclinical PET scanners. Data are taken from [5–15]

System	Recon. method	Crystal size (${\hbox {mm}}^{3}$)	FWHM (mm)^a^		
			*R*	*T*	*A*
SAFIR	FBP3DRP	$2.1\times 2.1\times 13.0$	2.77	1.89	2.83
SAFIR	MLEM	$2.1\times 2.1\times 13.0$	1.08	1.19	1.23
Hyperion II^D^	FBP^b^	$0.93\times 0.93\times 12$	1.7	1.8	1.4
NanoScan	SSRB FBP	$1.1\times 1.1\times 13.0$	1.50	1.32	0.91
MuPET	SSRB FBP	$1.24\times 1.4\times 9.5$	1.48	1.34	0.99
Inveon	FORE^c^ + 2D FBP	$1.5\times 1.5\times 10.0$	1.6	1.6	2.3
IRIS	MLEM	$1.6\times 1.6\times 12.0$	1.05	1.05	1.25
ClearPET	3D FBP	$2.0\times 2.0\times 10.0$	1.94	2.00	3.24
Mosaic HP	FBP3DRP	$2.0\times 2.0\times 10.0$	2.32	2.32	2.64
LabPET 8^TM^	SSRB FBP	$2.0\times 2.0\times 14.0$	1.65	1.70	1.40
LabPET 8^TM^	2D MLEM	$2.0\times 2.0\times 14.0$	1.0	1.0	1.7
microPET R4	FORE^b^FBP	$2.1\times 2.1\times 10.0$	2.13	2.21	2.72
Xtrim-PET	SSRB FBP	$2.1\times 2.1\times 10.0$	2.01	1.95	1.74
Bruker	MLEM	$50.0\times 50.0\times 10.0^{d}$	0.87	0.78	0.77

^a^FWHM at 5 mm radial offset in radial (R), tangential (T) and axial (A) directions.

^b^Hyperion II^D^ reports a spatial resolution of 0.9 mm FWHM in three directions, calculated using a Gaussian fit, at the center of the scanner for the MLEM reconstruction [36].

^c^Fourier rebinning algorithm [37].

^d^Monolithic crystals.

### Sensitivity

**Fig. 2:**
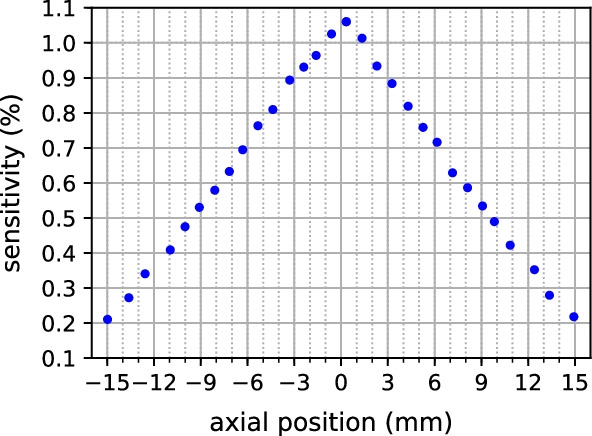
Sensitivity at different axial positions for the energy window of 391–601 keV.

Figure 2 shows the total sensitivity calculated for the point source data measured at different axial positions. The maximum sensitivity at the center is 1.06% for the energy window of 391–601 keV. It decreases to 0.2% at 15 mm axial offset. The expected triangular profile is clearly visible.

Table 3 compares the peak sensitivity of SAFIR and the reference scanners. The scanners have different axial FOVs and use different energy windows. Given its short axial FOV, the SAFIR prototype yields a high sensitivity which is in line with our goal for SAFIR.

**Table 3:** Comparison of the peak sensitivity of different preclinical PET scanners. Data are taken from [5–15]

System	Energy win. (keV)	Time win. (ns)	Axial FOV (mm)	Sensitivity (%)
SAFIR	391–601	0.5	36	1.06
SAFIR	250–650	0.5	36	2.57
Hyperion II^D^	250–625	200	96.7	4.0
NanoScan	250–750	5	94	8.4
MuPET	350–650	3.4	116	6.35
Inveon	350–625	3.4	127	6.72
IRIS	250–750	5.2	95	8
ClearPET	250–650	12	110	1.87
Mosaic HP	385–665	7	119	1.77
LabPET 8^TM^	250–650	20	75	1.33
microPET R4	350–650	6	78	2.4
Xtrim-PET	250–650	10	50.3	2.99
Bruker	–	–	150	11.0

### Count rate performance and scatter fraction

**Fig. 3:**
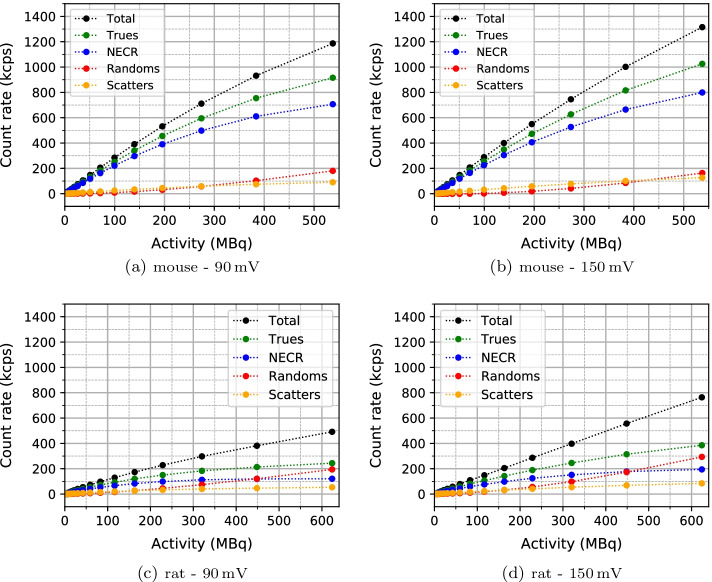
Count rate results for (**a**) the mouse scatter phantom, with relative timing threshold of 90 mV, (**b**) the mouse scatter phantom, with relative timing threshold of 150 mV, (**c**) the rat scatter phantom, with relative timing threshold of 90 mV and (**d**) the rat scatter phantom, with relative timing threshold of 150 mV. The highest activity for the mouse scatter phantom, 537 MBq, corresponds to an activity concentration of 2690 MBq/ml. The highest activity for the rat scatter phantom, 624 MBq, corresponds to an activity concentration of 1390 MBq/ml

Count rate results as a function of the activity in the phantom are plotted in Fig. 3, for the mouse and rat phantoms and for two relative timing thresholds of 90mV and 150 mV. The NECR peak is not reached for any of the measurements. This proves that the SAFIR prototype insert is capable of handling activities higher than 500 MBq.

The higher relative timing threshold of 150 mV leads to higher count rates and NECR. The highest NECR is 799 kcps at 537 MBq (corresponding to an activity concentration of 2690 MBq/ml) for the mouse scatter phantom and 121 kcps at 624 MBq (corresponding to an activity concentration of 1390 MBq/ml) for the rat scatter phantom. Due to the relatively short FOV of the SAFIR prototype, the detector receives many single γ-rays for the rat scatter phantom from outside the FOV, resulting in many randoms.

The scatter fractions are given in Table 4. They are in line with results obtained by others (Table 5).

**Table 4:** Scatter fractions (SFs) for the measurements of the mouse and rat scatter phantoms at two different relative timing threshold of 90 mV and 150 mV

Phantom	90 mV		150 mV	
	Mouse	Rat	Mouse	Rat
SF (%)	8.9	17.9	10.9	17.8

Table 5 presents the NECR peak and the scatter fraction of SAFIR and the *reference scanners*. Compared to other preclinical PET scanners, the SAFIR prototype shows an excellent count rate performance. All scanners reach their NECR peak at activities much less than 500 MBq. The SAFIR prototype has the highest measured NECR in comparison with other scanners with the exception of the Inveon [8] and MuPET [7] systems which have longer axial FOV and use larger energy windows.

**Table 5:** Comparison of NECR and scatter fraction (SF) of different preclinical PET scanners. Data are taken from [5–15]

System	Mouse phantom			Rat phantom		
	NECR peak (kcps)	Activity (MBq)$^{b}$	SF (%)	NECR peak (kcps)	Activity (MBq)	SF (%)
SAFIR^a^	799	537	10.9	121	624	17.8
Hyperion II^D^	407	46	13	–	–	–
NanoScan	406	30	17.3	119	25	34
MuPET	1100	57	11.9	352	65	28.0
Inveon	1670	131	7.8	592	110	17.2
IRIS	185	14	15.6	40	10	22.4
ClearPET	73	18	31	–	–	–
Mosaic HP	555	92	5.4	244	87	12.7
LabPET 8^TM^	279	82	15.6	94	91	29.5
microPET R4	168	91	18	89	81	28
Xtrim-PET	113.2	17	12.5	82.8	15	25.8
Bruker	486	23	–	240	23	–

^a^For SAFIR, the highest NECR values at the highest measured activities are reported as the NECR peaks are not reached

^b^Activity at which the NECR peak is measured

### Energy resolution and coincidence resolving time

**Fig. 4:**
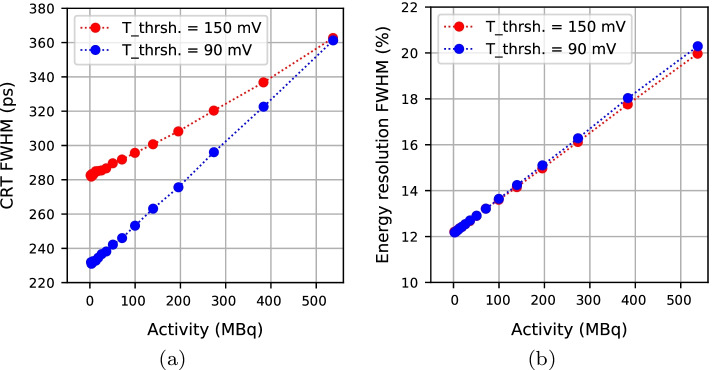
**a** Coincidence Resolving Time (CRT) and (**b**) energy resolution versus activity in the mouse scatter phantom measurement

The energy resolution and CRT versus activity are plotted in Fig. 4 for the mouse scatter phantom measurements. The pile-up increases with activity, thus energy resolution and CRT degrade by increasing activity. However, both values remain in a range fully sufficient for our application. The 90 mV relative timing threshold yields smaller CRTs than the 150 mV threshold, especially at lower activities.

### Image quality analysis

**Fig. 5:**
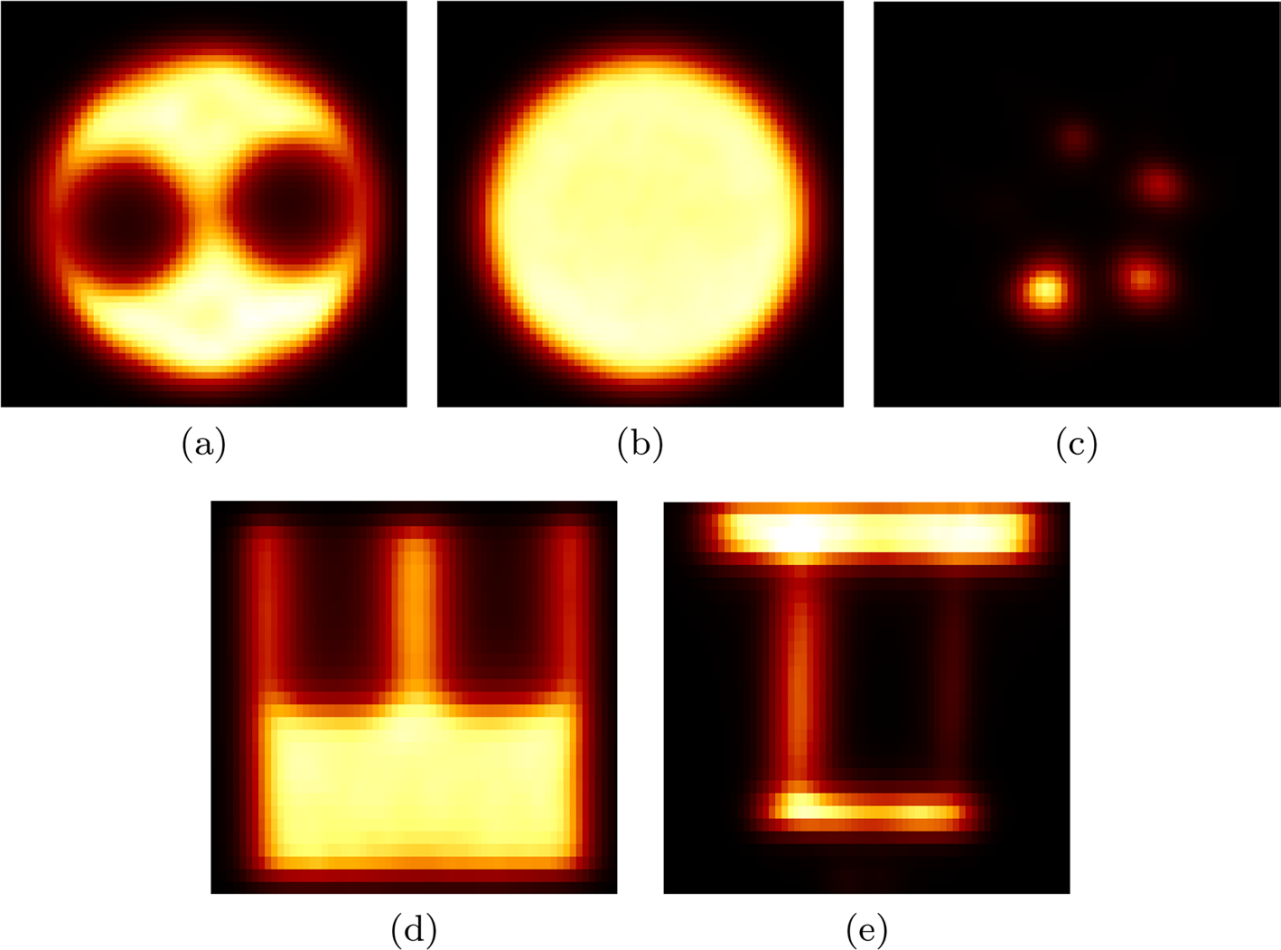
Different cross sections of the NEMA image quality phantom reconstructed using MLEM with 30 iterations, including random, scatter, attenuation and normalization corrections into the reconstruction. **a** Transverse view of cold rods, (**b**) transverse view of the uniform region, (**c**) transverse view of hot rods, (**d**) coronal view of cold rods and uniform region and (**e**) sagittal view of hot rods

Figure 5 shows transverse, coronal and sagittal cross sections of different regions in the NEMA image quality phantom. In short:

- All images are artifact-free.
- The uniform region has a 3.0% STD (Table 6).
- The Spill-Over Ratio for the water/air chamber is 0.18/0.17 (Table 7).
- The recovery coefficients for the smallest and largest rods are 0.13 and 0.88, respectively (Table 8).
- The smallest hot rod (1 mm diameter) is not visible in the image.

The deviation of the absolute voxel value in the uniform region is $4.6\%\pm 6.5\%$. For the uncertainty, we only propagated the uncertainties of the measurement of the activity and the volume of the image quality phantom.

The spill-over ratios of the air and water chambers are almost identical (0.01 difference), which is a direct result of including the data corrections into the reconstruction. Especially, the attenuation and scatter corrections influence the amount of background noise in the cold chambers with different attenuation properties.

**Table 6:** Uniformity measurement for the uniform region of the NEMA image quality phantom

Mean (count)	Minimum (count)	Maximum (count)	STD (%)	Deviation (%)
93,796	86,060	102,456	3.0	4.6

**Table 7:** Spill-Over Ratios (SORs) for the cold chambers in the NEMA image quality phantom

	SOR (%)	STD (%)
Air chamber	17.3	19.5
Water chamber	18.5	20.1

**Table 8:** Recovery coefficients for the hot rods in the NEMA image quality phantom

Rod diameter (mm)	Recovery coefficient	%STD
1	0.13	148.4
2	0.29	55.3
3	0.49	22.8
4	0.65	14.3
5	0.88	8.2

There is a strong correlation between the crystal size and the spatial resolution and thus with the recovery coefficient. SAFIR’s performance in terms of recovery coefficient is comparable with the scanners of similar crystal size (Table 9). However, scanners of smaller crystals such as [5, 8] and the Bruker scanner [15] with monolithic LYSO crystals outperform SAFIR.

Comparing Spill-Over Ratios is more difficult, as they depend on the reconstruction algorithm and whether or not attenuation and scatter corrections have been included into the reconstruction. Table 9 presents the Spill-Over Ratios and uniformities of SAFIR and the *reference scanners*. The uniformity achieved with the SAFIR prototype insert is the best among these reported in the table.

**Table 9:** Comparison of the recovery coeficient (RC) for the 3mm diameter rod, uniformity and Spill-Over Ratio (SOR) of different preclinical PET scanners. Data are taken from  [5–15, 38]

System	Recon. method	Corrected for AC/SC^b^	RC	STD (%)	SOR (%)	
				Uniform region	Water chamber	Air chamber
SAFIR	3D MLEM	yes/yes	0.49	3.0	17.3	18.5
Hyperion II^D^	3D MLEM	yes/yes	0.91	3.7	5.4	6.3
NanoScan	penalized MLEM	yes/yes	0.9	3.5	6.2	5.8
MuPET	FBP3DRP	yes/no	0.75	6.5	9	5
Inveon	FORE + 2D FBP	yes/yes	0.72	5.3	1.7	−0.6
IRIS	OSEM	yes/no	0.73	7	11	11
ClearPET	3D OSEM^a^	no/no	0.42	10.9	36.9	26.7
Mosaic HP	3D RAMLA^a^	yes/yes	0.56	5.1	6.3	2.7
LabPET 8^TM^	2D MLEM	no/no	0.58	7.0	20	11
microPET R4	FORE^a^ + 2D FBP	yes/no	0.60	4.5	6.2	4.6
Xtrim-PET	FORE + 2D OSEM	no/no	0.68	3.8	25	35
Bruker	3D MLEM	yes/no	0.91	4.5	6.2	4.6

^a^FORE = Fourier Rebinning; RAMLA = Row-Action Maximum-Likelihood Algorithm; OSEM = Ordered Subset Expectation Maximization

^b^ AC = Attenuation Correction; SC = Scatter Correction

## Conclusion

The performance of the SAFIR prototype insert has been evaluated according to the NEMA NU 4-2008 standard, while the insert was inside the 7 T MRI scanner without acquiring MRI data. The MRI-compatibility of the insert has been tested in previous studies [19, 27].

The results satisfy all requirements initially considered for the insert. The SAFIR prototype yields a high sensitivity for its short axial coverage. The count rate measurement results in an excellent NECR value of 799 kcps at the highest measured activity of 537 MBq using the mouse phantom, while not yet reaching the NECR peak. This demonstrates the prototype capability to handle high rate measurements, appropriate for dynamic imaging of fast biological processes. The spatial resolution has been shown to be as good as for other preclinical scanners with similar crystal size. The tests performed using the image quality phantom present a high uniformity and accuracy for the reconstructed images, suitable for quantitative PET imaging. In future studies, we plan to do more measurements beyond NEMA including the image quality phantom with 500 MBq activity.

The final version of the SAFIR insert is being developed with the same components as of the prototype but a quadrupled axial coverage, allowing dynamic whole body imaging of a mouse in a single bed position. This will also result in a higher sensitivity and NECR, especially for the rat scatter phantom as the contribution of the out-of-FOV $\gamma$-rays to the random count rate decreases.

## Data Availability

List mode data generated in this study can be provided upon request.
